# CT Characterization of Lipid Metabolism in Clear Cell Renal Cell Carcinoma: Relationship Between Liver Hounsfield Unit Values and Adipose Differentiation-Related Protein Gene Expression

**DOI:** 10.3390/ijms252312587

**Published:** 2024-11-23

**Authors:** Federico Greco, Andrea Panunzio, Laura Cerroni, Laura Cea, Caterina Bernetti, Alessandro Tafuri, Bruno Beomonte Zobel, Carlo Augusto Mallio

**Affiliations:** 1 Department of Radiology, Cittadella della Salute, Azienda Sanitaria Locale di Lecce, Piazza Filippo Bottazzi, 2, 73100 Lecce, Italy; 2 Research Unit of Radiology, Department of Medicine and Surgery, Università Campus Bio-Medico di Roma, Via Alvaro del Portillo, 21, 00128 Roma, Italy; laura.cerroni@alcampus.it (L.C.); laura.cea@unicampus.it (L.C.); c.bernetti@policlinicocampus.it (C.B.); b.zobel@policlinicocampus.it (B.B.Z.); c.mallio@policlinicocampus.it (C.A.M.); 3 Department of Urology, “Vito Fazzi” Hospital, Piazza Filippo Muratore, 1, 73100 Lecce, Italy; panunzioandrea@virgilio.it (A.P.); tafuri.alessandro@gmail.com (A.T.); 4 Fondazione Policlinico Universitario Campus Bio-Medico, Via Alvaro del Portillo, 200, 00128 Roma, Italy

**Keywords:** adipose differentiation-related protein, adipose tissue, clear cell renal cell carcinoma, computed tomography, hepatic steatosis, lipid metabolism, non-alcoholic fatty liver disease, radiogenomics

## Abstract

Radiogenomics is an emerging field that links imaging features with molecular characteristics of diseases. In clear cell renal cell carcinoma (ccRCC), metabolic reprogramming leads to lipid accumulation, influenced by the adipose differentiation-related protein (ADFP). This study aimed to investigate whether hepatic and tumoral Hounsfield Unit (HU) values could serve as noninvasive radiogenomic biomarkers for ADFP expression in ccRCC. We analyzed CT images of 185 ccRCC patients, comparing lipid-associated HU values in the liver and tumor across ADFP expression statuses. Patients with low-grade ccRCC expressing ADFP showed significantly lower minimum HU values in both liver and tumor tissue, indicating greater lipid accumulation. Additionally, ADFP expression correlated negatively with abdominal adipose tissue compartments and positively with minimum tumoral HU values, linking systemic lipid metabolism to tumor biology. These findings suggest that hepatic and tumoral HU measurements may serve as noninvasive markers of lipid accumulation related to ADFP, providing insight into metabolic alterations in ccRCC. While promising, these results require validation in larger, controlled studies due to sample size and variability limitations. This approach could enhance the radiogenomic assessment of ccRCC, supporting noninvasive insights into tumor metabolism and progression.

## 1. Introduction

Radiogenomics is a new area within radiology that links imaging features to the genomics of diseases [1,2]. These imaging phenotypes showcase broader molecular activities that can be identified through imaging techniques [1,2]. The field has been greatly influenced by the Human Genome Project, which has made genomic information widely available [3,4].

Metabolic reprogramming is the altered metabolism linked to the pathogenesis and progression of renal cell carcinoma (RCC) [5]. This reprogramming allows for fast-growing cancer cells to meet their increased demands for essential cellular components, such as DNA and membrane elements, while also producing higher amounts of molecules that support the enhanced energy requirements of the tumor [5]. Metabolic reprogramming, characterized by decreased tricarboxylic acid (TCA) cycle efficiency and activation of the von Hippel–Lindau (VHL)/hypoxia-inducible factor (HIF) pathway, leads to the increased expression of genes related to fatty acid (FA) synthesis and cholesterol (CHOL) production in cancer cells. These abundant resources generated through this process fuel the proliferation of cancer cells [6,7]. Clear cell renal cell carcinoma (ccRCC) tumors are rich in neutral lipids like CHOL esters and triglycerides, reflecting substantial lipid buildup within the cells [8,9]. The associated tumor growth and spread are strongly linked to FA synthesis, oxidative breakdown, and CHOL uptake and transport [10,11,12]. This significant lipid accumulation in ccRCC influences energy regulation and supplies lipid molecules essential for membrane formation during cancer cell proliferation [13]. As a result, targeting lipid buildup presents a promising therapeutic approach and helps uncover the intricate underlying mechanisms.

Adipose differentiation-related protein (ADFP), essential for FA uptake and lipid droplet formation, is significantly overexpressed in ccRCC at both the transcriptional and protein levels [14,15,16]. Elevated ADFP expression is also observed in adipocytes [17]. ADFP is a hypoxia-inducible gene, with its transcriptional activation controlled by HIFs [18]. The VHL protein (VHLp) forms a complex that facilitates HIF degradation; mutations in VHL result in its inactivation, preventing HIF degradation and thereby activating pro-angiogenic pathways and promoting cell growth [19,20,21,22]. This suggests that VHL mutations, by blocking HIF inactivation, may contribute to the upregulation of ADFP in ccRCC [16].

We now know that excess adipose tissue, especially visceral adipose tissue (VAT), plays an active role in the pathogenesis of RCC. In fact, increased VAT levels have been observed in cases of ccRCC [23].

When adipocytes receive insufficient oxygen, they trigger the release of HIF-1 from excess adipose tissue. This is accompanied by the abnormal secretion of adipokines such as leptin, adiponectin, resistin, and visfatin. This process could potentially connect obesity to the development of RCC [24,25,26].

It has been demonstrated that ccRCC patients with a low World Health Organization/International Society of Urological Pathology (WHO/ISUP) grade who express ADFP exhibit a significant decrease in the minimum tumoral HU values compared to those with a low WHO/ISUP grade but lacking ADFP expression [27]. Minimum tumoral HU values in ccRCC with a low WHO/ISUP grade with ADFP expression showed a negative correlation with VAT, subcutaneous adipose tissue (SAT), and total adipose tissue (TAT) [27].

Hepatic steatosis is the result of triglyceride accumulation in the liver, while the buildup of highly toxic free FAs driven by insulin resistance causes a large-scale release of free FAs from adipose tissue and increased de novo FA synthesis in the liver from glucose, which serves as the “initial hit” in the development of non-alcoholic fatty liver disease [28].

The aim of this study is to quantify, using a CT-based approach, lipid accumulation in the liver of patients with ccRCC expressing ADFP. The objective is to identify a potential radiogenomic biomarker of ADFP expression in ccRCC and to investigate its correlation with the lipid content within tumor cells and the amount of adipose tissue in the abdominal compartments in order to delineate the metabolic lipid profile through imaging in this patient population.

## 2. Results

Overall, 185 patients were included, of whom 125 were males, 109 (59.6%) harbored a localized disease (T1-2 stages), and 75 (40.5%) presented with low-grade (G1-2) tumors at final pathology (Table 1). Forty-two (22.7%) subjects had ADFP gene expression (Table 1).

Among patients with low-grade (G1-2) disease, 12 (16.0%) had ADFP gene expression (Table 2). These patients presented lower minimum tumoral HU values (−23, IQR: −38, −13 vs. −6, IQR: −16,3; *p* = 0.006) and lower minimum hepatic HU values (−4, IQR: −23, 10 vs. 16, IQR: 0,26; *p* = 0.039) compared to those without ADFP gene expression. No statistically significant differences emerged between groups regarding median abdominal tissue compartments values (Table 2). Among patients with high-grade (G3-4) tumors, 30 (27.3%) had ADFP gene expression. Here, no statistically significant differences emerged for abdominal tissue compartments, tumoral HU, and hepatic HU values between subjects who expressed ADFP and those who did not (data not showed).

**Figure 1:**
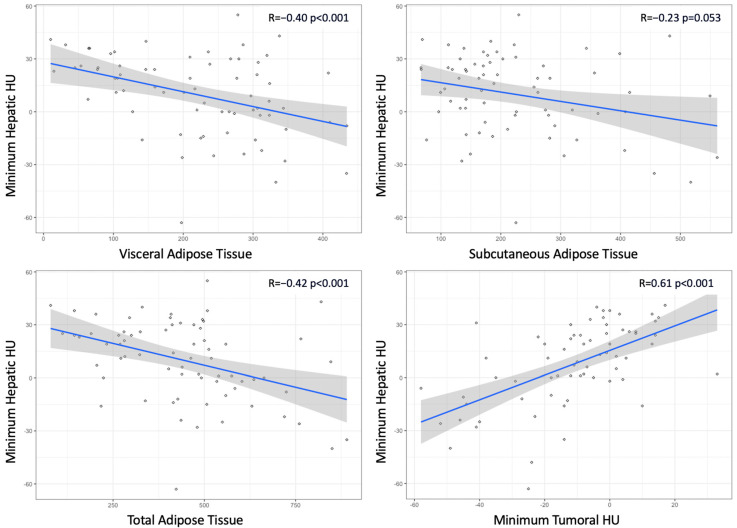
Scatterplots illustrating the relationship between hepatic minimum Hounsfield Units (HUs) and quantification of adipose tissue compartments and between hepatic and tumoral minimum HUs.

Focusing only on low-grade tumors, Figure 1 illustrates the relationship between minimum hepatic HU values and both quantification of adipose tissue compartments and minimum tumoral HU values. Accordingly, minimum hepatic HU values showed a negative correlation with median VAT, SAT, and TAT values. This correlation was stronger in patients who expressed ADFP compared to those who did not (R coefficient: −0.57 vs. −0.36 for VAT; −0.48 vs. −0.16 for SAT; −0.74 vs. −0.34 for TAT). Conversely, a positive correlation existed between hepatic and tumoral minimum HU values (R coefficient: 0.61).

## 3. Discussion

**Figure 2:**
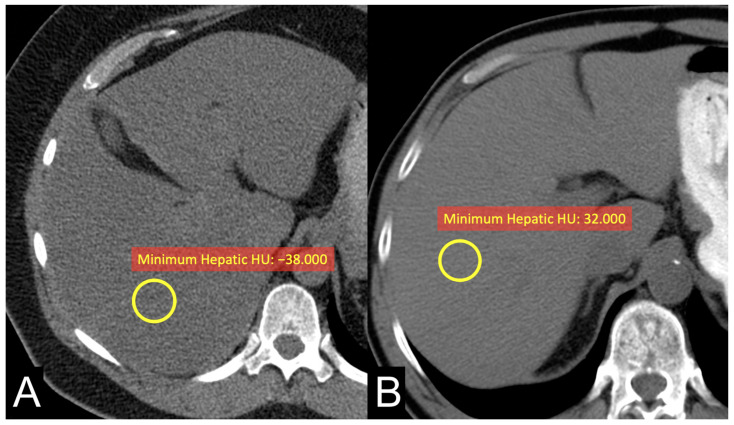
Unenhanced axial CT images of patients with low-grade ccRCC with ADFP expression (**A**) and low-grade ccRCC without ADFP expression (**B**) show yellow ROIs with different minimum hepatic HU values (HU −38 and 32, respectively).

**Figure 3:**
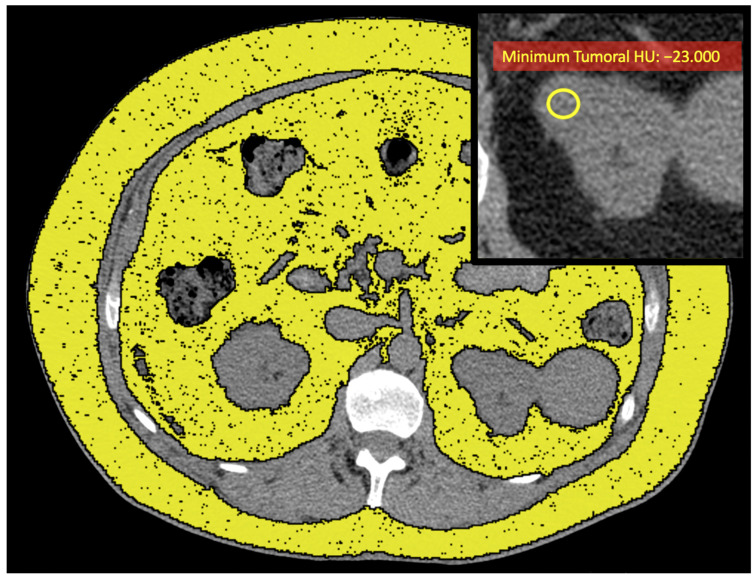
Unenhanced axial CT images of a patient with low-grade ccRCC with ADFP expression (same patient as in Figure 2A) show yellow ROIs highlighting the abdominal adipose tissue compartments, and the enlarged detail in the upper right shows the yellow ROI with a low minimum tumoral HU value (HU: −23).

The results revealed a significant association between ADFP expression and the decrease in minimum hepatic HU values (Figure 2) and minimum tumor HU values in patients with low WHO/ISUP grade ccRCC tumors. Specifically, patients with ADFP expression exhibited lower minimum hepatic HU values and lower minimum tumor HU values compared to those without ADFP expression (*p* = 0.039 and *p* = 0.006, respectively), indicating an increase in lipid deposition in the hepatocytes and tumor cells. Furthermore, we found a stronger negative correlation between minimum hepatic HU values and VAT, SAT, and TAT (*p* < 0.001 for VAT and TAT and *p* = 0.053 for SAT) and a stronger positive correlation with minimum tumoral HU values (*p* < 0.001) in ccRCC patients with ADFP expression (Figure 3) compared to patients without ADFP expression.

This suggests that ADFP expression is associated not only with lipid accumulation in tumor cells but also with alterations in hepatic lipid metabolism, potentially linking the two through a common metabolic pathway. These findings highlight the potential of hepatic and tumor HU values as noninvasive radiogenomic biomarkers to identify lipid metabolic changes associated with ADFP expression in ccRCC.

A reliable estimate of intracellular lipid content in the liver can be obtained using HU values. Kim et al. assessed whether HU values from CT scans can reliably diagnose mild hepatic steatosis and assess the severity of steatosis and intrahepatic inflammation. The results showed that HU metrics effectively diagnosed mild steatosis and correlated with steatosis and inflammation severity. CT-derived HU values proved useful for stratifying liver fat content and inflammation, making them promising noninvasive tools [29].

It has been demonstrated that ccRCC patients with low WHO/ISUP grade show a reduction in minimum tumoral HU values compared to those with the same WHO/ISUP grade but without ADFP expression [27]. Additionally, a notable decrease in minimum tumoral HU values was observed in ccRCC patients with a low WHO/ISUP grade and ADFP expression compared to those with a high WHO/ISUP grade and ADFP expression [27]. A negative correlation was also identified between minimum tumor HU values and VAT, SAT, and TAT in ccRCC patients both with and without ADFP expression, with a stronger association in those expressing ADFP [27]. This suggests that there is a significant link between metabolic lipid CT features and ADFP expression in ccRCC patients [27]. The observation of a notable reduction in minimum tumoral HU values among ccRCC patients with a low WHO/ISUP grade and ADFP expression, compared to those without ADFP expression, highlights the importance of intracellular lipid accumulation in this context [27]. In ccRCC, lower HU values typically indicate a higher lipid content within the tumor and may reflect the phenotypic counterpart of ADFP activity involved in FA uptake and lipid droplet formation [27]. Similarly, the low minimum HU values detected in the liver reflect greater lipid accumulation in hepatocytes. This suggests that ADFP expression may be linked to altered lipid metabolism in both the liver and tumor tissue in ccRCC patients, indicating a potential common metabolic pathway between hepatic tissue and tumor cells. CcRCC is notable for its elevated levels of lipids, including CHOL, CHOL esters, and phospholipids within the cytoplasm. This lipid buildup gives the tumor a characteristic yellow color on gross examination, and, under routine hematoxylin–eosin staining, it primarily consists of ‘clear cells’ [30,31]. In particular, in ccRCC cases with a lower nuclear grade (G1 and G2), a typical ‘clear cell’ appearance is observed. However, with an increase in nuclear grade (G3 and G4), the cytoplasm takes on a more eosinophilic quality, and the ‘clear cell’ characteristic becomes less prominent [31]. Yao et al. found that low-grade ccRCCs generally show increased ADFP mRNA and protein expression relative to high-grade tumors [16,32]. These observations suggest that ADFP overexpression plays a role in increased lipid absorption and storage in low-grade ccRCC when compared to high-grade tumors [32]. Additionally, ADFP expression levels appear to correlate with the macroscopic and microscopic morphological characteristics of ccRCC [32].

It is worth noting that lower minimum hepatic HU values serve as an additional radiogenomic biomarker for ADFP expression in ccRCC patients with a low WHO/ISUP grade. This connection can be argued by considering how lower hepatic HU values mirror increased lipid accumulation in hepatocytes, similar to the lipid buildup seen in ccRCC tumor cells. Since ADFP expression is linked to lipid uptake and storage, these low hepatic HU values could indicate a broader metabolic impact beyond the tumor itself, possibly due to a shared lipid processing pathway between adipose, hepatic, and tumor tissues. Thus, lower hepatic HU values could offer a noninvasive means of predicting ADFP expression and, by extension, lipid metabolic activity within ccRCC cells. This approach could enhance patient assessment by providing insights into tumor biology and metabolic changes through imaging biomarkers, which might be valuable for characterizing tumors and potential behavior without invasive procedures.

These findings suggest that systemic lipid metabolism is closely linked to the tumor’s biological behavior. Clinically, these data could support the integration of radiogenomic measurements (such as hepatic and tumoral HU values) into monitoring protocols to identify patients with significant metabolic alterations, potentially correlated with different disease courses or therapeutic responses. Furthermore, the correlations between ADFP and adipose compartments could be useful for risk stratification, considering lipid metabolism as a potential therapeutic target, particularly in patients with low WHO/ISUP grade ccRCC. Targeted therapies aimed at inhibiting HIF could also have effects on the expression of ADFP [33]. Finally, the involvement of lipid metabolism in ccRCC suggests that targeted interventions, such as personalized therapeutic strategies associated with diets, could improve clinical outcomes. However, to confirm the clinical applicability of these observations, future studies with larger and more diverse cohorts are needed.

ADFP plays an active role in the pathogenesis of several cancers. Meng et al. investigated the role of ADFP in lung adenocarcinoma by measuring its levels in the serum of lung cancer and benign disease patients. They found that ADFP was highly expressed in the serum of lung cancer patients, particularly those with lung adenocarcinoma. Using shRNA to knock down or overexpress ADFP in A549 and NCI-H1299 cells, the authors demonstrated that ADFP promoted cell proliferation and increased the p-Akt/Akt ratio in vitro. In vivo, ADFP enhanced tumor formation in nude mice, with elevated levels of p-Akt/Akt, Ki67, and PCNA. The inhibition of Akt phosphorylation with MK-2206 reduced cell proliferation, which was reversed in ADFP-overexpressing cells. However, ADFP did not affect invasion, migration, or adhesion in lung adenocarcinoma cells. These results suggest that ADFP promotes lung adenocarcinoma cell proliferation through increased Akt phosphorylation [34]. Hayakawa et al. investigated the accumulation of lipid droplets and the expression of ADFP in follicular thyroid carcinoma cells. They found that cultured follicular thyroid carcinoma cells (FTC-133 and RO82W-1) had increased lipid droplet populations compared to normal thyroid follicular cells. Treatment with PI3K/Akt/mTOR pathway inhibitors reduced the accumulation of lipid droplets by downregulating the PI3K/Akt/mTOR/SREBP1 signaling pathway. Immunocytochemistry revealed ADFP expression in the lipid droplets of FTC-133 cells. Additionally, the immunohistochemical analysis of resected human thyroid tissues showed significantly higher ADFP expression in follicular thyroid carcinoma compared to follicular thyroid adenoma and adjacent non-tumorous tissue. These findings suggest that the evaluation of ADFP expression can help distinguish follicular thyroid carcinoma from follicular thyroid adenoma in surgical specimens [35].

Our study employs a robust statistical methodology, including the Wilcoxon rank sum tests and Fisher’s exact tests. However, different alternative statistical methodologies that may incorporate regression models or machine learning approaches for predictive analyses can be used for future studies.

We recognize several limitations in this study that may influence both the interpretation and applicability of its findings. First, the limited sample size could reduce this study’s statistical robustness and the reliability of the detected associations. Potential confounding factors, such as the influence of other genes involved in lipid droplet accumulation—namely Ancient Ubiquitous Protein 1 (AUP1) and Acyl-CoA Synthetase 3 (ASCL3)—were not thoroughly controlled for in this analysis [36,37]. Additionally, the presence of potential confounding factors, such as diet, medication, and preexisting liver conditions, can influence hepatic lipid accumulation by altering lipid metabolism pathways, modulating inflammatory responses, or affecting liver function, thereby potentially skewing this study’s findings [38].

To clarify ADFP’s role in ccRCC, future studies with larger cohorts and minimal gene expression confounders would be valuable. The retrospective nature of this study may also introduce selection bias and limits the ability to infer causal links between ADFP expression and imaging characteristics. Moreover, variations in imaging equipment and protocols across different centers were not addressed, potentially impacting the reproducibility and consistency of the results. Implementing standardized imaging techniques could help reduce this variability in future research. Patient-specific biological differences, including diversity in tumor characteristics, metabolic profiles, and risk factors, could further complicate the observed relationships between ADFP expression, imaging biomarkers, and clinical outcomes. This study did also not account for certain variables that might influence the link between ADFP expression and imaging parameters, such as comorbid conditions, medications, or lifestyle factors, which could restrict the scope of the analysis. Given these limitations, these findings should be interpreted cautiously, and additional studies addressing these concerns are necessary to enhance the evidence in this field.

## 4. Materials and Methods

### 4.1. Lipid Metabolism Imaging Features

The evaluation of imaging features representing lipid metabolism in ccRCC was carried out by measuring hepatic Hounsfield Units (HUs), tumor HUs, and quantifying abdominal adipose tissue compartments using Horos v.4.0.0 RC2 software. Hepatic HU values were assessed on unenhanced scans by placing a region of interest (ROI) in the liver parenchyma and recording the minimum, maximum, and mean HU values within that area. Similarly, tumor HUs were evaluated on unenhanced images by positioning an ROI within the solid component of the tumor and collecting the minimum, maximum, and mean HU values from that region. The ROI was subsequently investigated with a smaller ROI (equal to 5%) to verify that these values were included in at least 5% of the ROI. These measurements allowed for an estimation of lipid content within both hepatocytes and tumor cells. Since adipose tissue typically exhibits HU values ranging from −50 to −100, lower HU values in the liver parenchyma and tumor’s solid component indicate a greater accumulation of intracellular lipids [27,39].

TAT, VAT, and SAT were measured using a semi-automatic tool within Horos v.4.0.0 RC2 software. This tool enabled the analysis of all cross-sectional CT images by selecting the HU range specific to adipose tissue. The results were expressed as areas (cm²) based on a single axial image, taken 3 cm above the lower border of the L3 vertebra, following a previously established protocol [40].

All ROIs were determined by a consensus between two radiologists (F.G. with 9 years of experience and C.A.M. with 13 years of experience), both of whom were blinded to the patients’ clinical information.

### 4.2. Statistical Analysis

Three sets of analyses were performed. First, we tabulated demographics and clinical–pathological characteristics of the entire patient population. Descriptive statistics included frequencies and proportions for categorical variables; medians and interquartile ranges (IQRs) were reported for continuously coded variables. Second, abdominal tissue compartments (VAT, SAT, and TAT median values), tumoral HU (median, minimum, and maximum values), as well as hepatic HU (median, minimum, and maximum values) were compared according to ADFP gene expression (yes vs. no) and tumor grade (low [G1-2] vs. high [G3-4]). Here, the Wilcoxon rank sum test and Fisher’s exact test examined the statistical significance of differences in medians and proportions, respectively. Finally, correlation analysis was used to test the relationship between minimum hepatic HU values, the quantification of abdominal adipose tissue compartments, and minimum tumoral HU values. Here, Pearson’s correlation coefficient was used if values were sampled from the normal population; otherwise, the Spearman correlation coefficient was used. All tests were two-sided with a level of significance set at *p* < 0.05. The R software environment for statistical computing and graphics (version 4.1.2, R foundation for Statistical Computing, Vienna, Austria) was used for all analyses.

## 5. Conclusions

This study demonstrates that ADFP expression in low-grade ccRCC is associated with increased lipid accumulation in both tumor cells and the liver, reflected by lower minimum HU values in these tissues. Our conclusion indicates that both hepatic and tumoral HU values could serve as noninvasive biomarkers; however, due to limited sample sizes, we must exercise greater caution when interpreting our findings. Performing an in-depth comparison with similar large-scale studies could shed more light on what limitations exist while simultaneously validating its approach.

## Figures and Tables

**Table 1:** Descriptive characteristics of the study population.

	**Overall** n = 185 ^1^
**Sex (Males)**	125 (67.6%)
**Primary tumor size** (mm)	54.0 (38.0, 82.0)
**Tumor grade** (Fuhrman) Low-grade (G1-2) High-grade (G3-4)	75 (40.5%) 110 (59.5%)
**Tumor stage** Stage I Stage II Stage III Stage IV	92 (50.3%) 17 (9.3%) 48 (26.2%) 26 (14.2%)
**ADFP expression**	42 (22.7%)
**Abdominal adipose tissue compartments** VAT SAT TAT	209.7 (110.2, 284.5) 184.8 (138.2, 275.6) 413.0 (285.8, 512.5)
**Tumoral HUs** Median Minimum Maximum	35 (30, 40) −5 (−18, 3) 77 (67, 91)
**Hepatic HUs** Median Minimum Maximum	59 (51, 65) 11 (−6, 27) 106 (92, 122)

^1^ Median (IQR); n (%). Abbreviations: ADFP, adipose differentiation-related protein; VAT, visceral adipose tissue; SAT, subcutaneous adipose tissue; TAT, total adipose tissue; HUs, Hounsfield Units.

**Table 2:** Quantification of adipose tissue compartments, tumoral, and hepatic HU values in patients with low-grade (G1-2) tumors according to ADFP gene expression.

	No ADFP Expression n = 63 (84.0%) ^1^	ADFP Expression n = 12 (16.0%) ^1^	*p*-Value ^2^
**Abdominal adipose tissue compartments**			
VAT	220 (109, 302)	266 (220, 329)	0.12
SAT	186 (141, 277)	225 (153, 349)	0.4
TAT	439 (282, 527)	481 (420, 722)	0.2
**Tumoral HUs**			
Median	32 (27, 37)	30 (24, 32)	0.10
Minimum	−6 (−16, 3)	−23 (−38, −13)	**0.006**
Maximum	74 (62, 87)	71 (63, 100)	0.5
**Hepatic HUs**			
Median	60 (49, 64)	62 (48, 66)	0.9
Minimum	16 (0, 26)	−4 (−23, 10)	**0.039**
Maximum	104 (90, 122)	108 (100, 130)	0.2

^1^ Median (IQR); n (%) ^2^ Wilcoxon rank sum test; Fisher’s exact test. Abbreviations: ADFP, adipose differentiation-related protein; VAT, visceral adipose tissue; SAT, subcutaneous adipose tissue; TAT, total adipose tissue; HUs, Hounsfield Units.

## Data Availability

The data presented in this study are openly available in The Cancer Imaging Archive (https://www.cancerimagingarchive.net/collection/tcga-kirc/ accessed on 1 November 2019).
